# Effect of aclidinium bromide on cough and sputum symptoms in moderate-to-severe COPD in three phase III trials

**DOI:** 10.1136/bmjresp-2016-000148

**Published:** 2016-12-08

**Authors:** Lorcan McGarvey, Alyn H Morice, Jaclyn A Smith, Surinder S Birring, Ferran Chuecos, Beatriz Seoane, Diana Jarreta

**Affiliations:** 1 Centre for Infection and Immunity, Queen's University Belfast, Belfast, UK; 2 Hull York Medical School, University of Hull, Castle Hill Hospital, Cottingham, UK; 3 Centre for Respiratory Medicine and Allergy, University of Manchester, University Hospital of South Manchester, Manchester, UK; 4 Division of Asthma, Allergy and Lung Biology, King's College London, London, UK; 5 R&D Centre, AstraZeneca PLC, Barcelona, Spain

**Keywords:** COPD Pharmacology, Cough/Mechanisms/Pharmacology

## Abstract

**Background:**

Cough and sputum are troublesome symptoms in chronic obstructive pulmonary disease (COPD) and are associated with adverse outcomes. The efficacy of aclidinium bromide 400 µg twice daily in patients with stable COPD has been established in two phase III studies (ACCORD COPD I and ATTAIN) and a phase IIIb active-comparator study. This analysis evaluated cough-related symptoms across these studies.

**Method:**

Patients were randomised to placebo, aclidinium 200 µg or 400 µg twice daily in ACCORD (12 weeks) and ATTAIN (24 weeks), or to placebo, aclidinium 400 µg twice daily or tiotropium 18 µg once daily (6-week active-comparator study). Analysed end points included changes from baseline in Evaluating Respiratory Symptoms (E-RS; formerly known as EXAcerbations of Chronic pulmonary disease Tool), total and cough/sputum scores and frequency/severity of morning and night-time cough and sputum symptoms.

**Results:**

Data for 1792 patients were evaluated. E-RS cough/sputum domain scores were significantly reduced with aclidinium 400 µg versus placebo in ATTAIN (−0.7 vs −0.3, respectively; p<0.01) and the active-comparator study (−0.6 vs −0.2, respectively; p<0.01). In the active-comparator study, significantly greater improvements were observed with aclidinium versus placebo for severity of morning cough (−0.19 vs −0.02; p<0.01) and phlegm (−0.19 vs −0.02; p<0.05). In ACCORD, aclidinium reduced night-time cough frequency (−0.36 vs 0.1 for placebo; p<0.001) and severity (−0.24 vs −0.1 for placebo; p<0.05), and frequency of night-time sputum production (−0.37 vs 0.05 for placebo; p<0.001).

**Conclusions:**

Aclidinium 400 µg twice daily improves cough and sputum expectoration versus placebo in stable COPD.

**Trial registration numbers:**

NCT00891462; NCT01001494; NCT01462929.

**Key messages**

- Cough and sputum in COPD have a substantial impact on patients' health status, yet there are relatively few studies that have investigated the effect of bronchodilators on these symptoms.
- In this paper, we analyse data from three Phase III studies to elucidate the effect of aclidinium bromide on cough and sputum. The results suggest that in addition to improving lung function, LAMAs, such as aclidinium, can improve cough and sputum expectoration compared with placebo in patients with COPD.
- As cough and sputum impact negatively on overall patient wellbeing, controlling these symptoms may represent an important additional therapeutic benefit of this class of drugs.

## Introduction

Chronic obstructive pulmonary disease (COPD) is characterised by persistent and progressive airflow limitation and an enhanced inflammatory response to noxious stimuli.^1^ The resulting lung injury leads to breathlessness and other characteristic symptoms of COPD, including cough and sputum.

In patients with COPD, chronic cough and sputum production are associated with lung-function decline,^2^ more frequent exacerbations and hospitalisations, and increased risk of death.^3^ ^4^ Accumulation of mucus in small airways is also associated with disease progression,^5^ and a productive cough has been shown to be independently associated with increased mortality in smokers with mild-to-moderate airflow obstruction.^6^ Cough symptoms also impact adversely on the health status of patients with COPD to a similar degree to that observed in bronchiectasis, asthma and chronic cough.^7^ The importance of cough and sputum symptoms in defining a patient's overall well-being is reflected in the inclusion of these items in the COPD Assessment Test, a patient-reported outcomes tool designed to assess overall COPD-related health status.^8^ ^9^

Between disease exacerbations, when COPD is considered stable, there may still be marked daily variability in patients' perceptions of symptom severity. In a pan-European cross-sectional study in patients with COPD, cough and phlegm were reported to be most troublesome in the morning.^10^ However, in a recent observational study of COPD symptoms, despite overall night-time symptoms being less prevalent than in the morning and during the day, cough was still the most common symptom at night.^11^

Despite the evidence of a clear association between cough and adverse clinical outcomes, its significance in patients with COPD is often underappreciated.^12^ ^13^ In addition, almost nothing is known about the effect of current first-line COPD treatments on symptoms of cough and sputum, and the need for studies to address this has been highlighted.^12^ Aclidinium bromide is a long-acting muscarinic antagonist (LAMA) that inhibits the action of acetylcholine at M3 receptors in the lungs, indirectly leading to airway smooth muscle relaxation. Aclidinium is approved as a maintenance bronchodilator treatment in patients with COPD.^14–16^ Several phase III studies have shown that aclidinium 400 µg twice daily improves lung function and symptoms in patients with moderate-to-severe airflow limitation.^17–23^ In this manuscript, we report our analysis of the data from three of these studies, ACCORD COPD I, ATTAIN and a 6-week active-comparator study, which was undertaken to determine the effect of the approved dose of aclidinium (400 µg twice daily metered dose; equivalent to aclidinium 322 μg delivered dose) on cough and sputum symptoms in patients with stable moderate-to-severe COPD. The three phase III studies reported here were selected on the basis that they had similar inclusion/exclusion criteria and included end points that assessed the efficacy of aclidinium 400 µg twice daily on cough and sputum symptoms. Four additional phase III studies of aclidinium did not record cough data so could not be included in this analysis.

## Methods

In these analyses, only data from patients randomised to placebo, aclidinium 400 µg twice daily (the dose approved for use in patients with COPD) or tiotropium 18 µg once daily (also the approved dose) were evaluated. The purpose of this additional analysis was to assess the impact of aclidinium on cough and sputum symptoms across three clinical studies, including the relationship between symptoms and time of day. All end points were preplanned, with the exception of post hoc analyses assessing the correlation between Evaluating Respiratory Symptoms (E-RS; formerly known as EXAcerbations of Chronic pulmonary disease Tool) cough and sputum domain score and cough severity score in the active-comparator study and change from baseline in E-RS total and cough and sputum domain scores in patients who had ≥1 exacerbation event in the ATTAIN study.

### Study design

ACCORD COPD I (ClinicalTrials.gov identifier: NCT00891462) and ATTAIN (ClinicalTrials.gov identifier: NCT01001494) were multinational, randomised, double-blind, placebo-controlled phase III studies.^17^ ^18^ Following screening and a 2-week run-in period, patients were randomised (1:1:1) to receive aclidinium 200 µg, aclidinium 400 µg (metered dose; equivalent to aclidinium 322 µg delivered dose) or placebo twice daily via the Genuair™/Pressair^®i^ inhaler for 12 weeks in ACCORD COPD I and 24 weeks in ATTAIN.

The third study was a randomised, double-blind, double-dummy, placebo-controlled and active-controlled phase IIIb study (ClinicalTrials.gov identifier: NCT01462929).^19^ Following a 2–3-week run-in period, patients were randomised (2:2:1) to receive aclidinium 400 µg twice daily (metered dose; equivalent to aclidinium 322 µg delivered dose), tiotropium 18 µg once daily in the morning via HandiHaler^®^ or placebo for 6 weeks.

In all three studies, inhaled albuterol/salbutamol (108/100 µg/puff) was permitted as relief medication as long as it was discontinued 6 hours prior to study visits. Additional permitted medications included inhaled corticosteroids, oral or parenteral corticosteroids (≤10 mg/day of prednisone or 20 mg every other day), oral sustained-release theophyllines and oxygen therapy (<15 hours/day), provided that treatment was stable for ≥4 weeks before screening. Other long-acting bronchodilators and anticholinergic drugs were washed out prior to screening and were not allowed during the treatment periods.

All studies were conducted in accordance with the Declaration of Helsinki, International Conference on Harmonisation/Good Clinical Practice Guidelines and local regulations. The protocols were approved by institutional review boards/independent ethics committees at each site, and all patients gave written informed consent.

### Study populations

Detailed inclusion/exclusion criteria for the three studies have been reported previously.^17–19^ Briefly, each study enrolled male and female patients (≥40 years old) with a diagnosis of stable COPD and moderate-to-severe airflow obstruction (postbronchodilator forced expiratory volume in 1 s (FEV_1_) ≥30% and <80% of the predicted value and FEV_1_/forced vital capacity ratio <70%)^1^ who were current or former smokers with a smoking history of ≥10 pack-years. The presence of cough or sputum symptoms at baseline was not a specific inclusion criterion in any of the studies.

Exclusion criteria included any respiratory tract infection or COPD exacerbation within 6 weeks prior to screening (3 months if exacerbation resulted in hospitalisation), any clinically relevant respiratory conditions, including a history or current diagnosis of asthma and a history of hypersensitivity to inhaled anticholinergics or other inhaled medications.

### Study assessments

A summary of patient-reported outcome measures used to capture symptoms in each clinical trial and corresponding end points are shown in online supplementary table S1. Baseline values for all end points were calculated as the average scores over the 2–3-week screening period prior to randomisation.

10.1136/bmjresp-2016-000148.supp1 supplementary tables

#### Daily symptoms

In ATTAIN and the active-comparator study, daily respiratory symptoms were assessed using the E-RS algorithm.^24–26^ The EXACT is a 14-item electronic daily diary used to quantify and measure exacerbations of COPD. It is completed by patients at night with a recall period of ‘today’ and captures symptoms of COPD including cough and sputum production. The E-RS total score (range 0–40) is a derivative tool which uses the 11 EXACT items that relate specifically to respiratory symptoms, with higher scores indicating more severe symptoms; the E-RS cough and sputum domain score is the sum of the three EXACT items that relate specifically to cough and sputum symptoms (range 0–11). Responder criteria for E-RS total score and E-RS cough and sputum domain scores have been proposed as a change of ≥−2.0 units in the E-RS total score and ≥−0.7 in the E-RS cough and sputum domain score.^27^ The E-RS was not used in the ACCORD study.

#### Morning and night-time cough and sputum symptoms

Cough and sputum symptoms during the morning and night-time were assessed in the three phase III studies using questionnaires developed by the study sponsors.^28^ ^29^ In ATTAIN, a 6-item night-time and morning symptoms of COPD questionnaire, completed by patients at approximately the same time every morning using an electronic patient diary, was used to assess the number of days patients experienced a range of morning or night-time symptoms, including coughing and bringing up phlegm or mucus. The questionnaire included one item that asked patients if they experienced symptoms during the night and one item that asked about their symptoms since they got out of bed to start the day.

In the active-comparator study, morning symptoms were assessed using a 9-item COPD symptom questionnaire, completed daily by patients between 7:00 am and 11:00 am using an electronic diary. Early morning was defined as the time from when patients got out of bed to start the day until they started their daily activities. One item of the questionnaire was related to the presence of a range of early-morning symptoms, including cough and phlegm, with five items related to the severity of these symptoms. Patients assessed the severity of their overall morning symptoms (5-point scale: 1=‘I did not experience any symptoms’; 2=‘mild’; 3=‘moderate’; 4=‘severe’; 5=‘very severe’) and the severity of individual symptoms, including cough and difficulty bringing up phlegm (5-point scale: 0=‘no symptoms’; 1=‘mild’; 2=‘moderate’; 3=‘severe’; 4=‘very severe’).

In ACCORD COPD I, night-time symptoms were assessed using an 11-item COPD night-time symptoms questionnaire, adapted from an existing COPD symptom questionnaire^30^ to include additional items assessing the frequency of COPD symptoms, such as night-time breathlessness, cough, sputum production and wheezing, during the previous night. Patients completed the questionnaire daily in the morning using an electronic patient diary (the recall period was ≤24 hours). The frequency of night-time symptoms was assessed on a 5-point scale: 0=‘never’; 1=‘1–2 times’; 2=‘3–4 times’; 3=‘5–6 times’; 4=‘7 or more times’. The severity and impact of night-time symptoms were assessed on a 5-point scale: 0=‘no symptoms’; 1=‘symptoms present but caused little/no discomfort’; 2=‘mild symptoms that were unpleasant but caused little/no discomfort’; 3=‘moderate symptoms that caused discomfort but did not affect daily activities’; 4=‘severe symptoms that interfered with normal daily activities’.

### End points

Predefined efficacy end points included: changes from baseline in E-RS total score and E-RS cough and sputum domain score over the study period (ATTAIN and active-comparator study); the percentage of days with morning or night-time symptoms over the study period (ATTAIN); changes from baseline in the percentage of days without morning symptoms and the severity of morning cough and difficulty bringing up phlegm over the study period (active-comparator study) and changes from baseline at week 12 in COPD night-time symptoms (ACCORD COPD I). To investigate the reliability of different measures of cough symptoms used in these analyses, a post hoc analysis assessed the correlation between changes from baseline in E-RS cough and sputum domain scores in those patients who had ≥1 exacerbation event in the ATTAIN study, and the severity of morning cough (based on the symptom questionnaires) at week 6 in the active-comparator study. These were selected as both measures assess the improvement from baseline in symptom severity.

Safety and tolerability were assessed in all three studies by recording adverse events. Additional safety assessments included a physical examination, laboratory tests, vital signs and ECGs.

### Statistical analyses

Demographic and baseline characteristics were assessed in the intent-to-treat (ITT) population (all treated patients who had baseline and at least one postbaseline FEV_1_ assessment) and are reported as mean (SD) or percentage, as appropriate. Efficacy analyses were performed in the ITT population. Changes from baseline in E-RS total and cough and sputum domain scores (ATTAIN and active-comparator study), percentage of days with morning or night-time cough symptoms (ATTAIN) and changes from baseline in the percentage of days without morning symptoms and the severity of morning symptoms (active-comparator study) were analysed using an analysis of covariance (ANCOVA) model, with treatment group and sex as factors and age and corresponding baseline as covariates. Changes from baseline in the frequency and severity of night-time symptoms (ACCORD COPD I) were analysed using an ANCOVA model with treatment as a factor and the corresponding baseline as a covariate. Data are reported as least squares mean (SEM), least squares mean differences (95% CIs) or percentages, as appropriate. For the post hoc analysis, Pearson coefficients were used to evaluate the correlation between improvements in E-RS cough and sputum domain score and the scores from the cough severity question in symptom questionnaires.

Additional post hoc analyses assessed the change from baseline in E-RS total and cough and sputum domain scores in those patients who had ≥1 exacerbation event identified using the EXACT in the ATTAIN study. An EXACT-identified event was defined as a persistent increase from baseline in total EXACT score of ≥9 points for ≥3 days or ≥12 points for ≥2 days.^26^ ^31^

## Results

### Patient population

The ITT populations in ACCORD COPD I, ATTAIN and the active-comparator study included 559, 819 and 414 patients, respectively. Demographics and baseline clinical characteristics of the study populations have been reported previously;^17–19^ the demographics and baseline clinical characteristics in the placebo, aclidinium 400 μg and tiotropium arms are shown in table 1. E-RS scores and symptom questionnaire scores at baseline in the placebo, aclidinium 400 μg and tiotropium study arms are shown in online supplementary table S2.

**Table 1:** Demographics and baseline clinical characteristics (ITT population)

	ACCORD COPD I		ATTAIN		Active-comparator study		
Characteristic	Placebo (N=185)	Aclidinium 400 µg twice daily (N=190)	Placebo (N=273)	Aclidinium 400 µg twice daily (N=269)	Placebo (N=85)	Aclidinium 400 µg twice daily (N=171)	Tiotropium 18 µg once daily (N=158)
Age (years), mean (SD)	65.0 (9.2)	64.9 (9.5)	62.0 (8.0)	62.9 (8.4)	62.2 (8.2)	61.8 (8.2)	62.8 (7.9)
Gender (male), n (%)	95 (51.4)	100 (52.6)	189 (69.2)	182 (67.7)	48 (56.5)	114 (66.7)	116 (73.4)
Current smoker, n (%)	87 (47.0)	80 (42.1)	144 (52.8)	148 (55.0)	47 (55.3)	93 (54.4)	84 (53.2)
Smoking history (pack-years), mean (SD)	52.9 (28.1)	57.2 (28.5)	38.9 (18.3)	41.7 (21.1)	39.6 (15.4)	41.5 (22.4)	45.0 (21.8)
Postbronchodilator FEV_1_,* mean (SD), L	1.6 (0.6)	1.5 (0.5)	1.6 (0.5)	1.6 (0.5)	1.6 (0.5)	1.6 (0.5)	1.7 (0.5)
Postbronchodilator FEV_1_% predicted,* mean (SD)	54.7 (13.4)	54.1 (12.9)	56.6 (12.8)	56.2 (12.2)	55.5 (11.8)	55.8 (13.3)	56.0 (13.2)
Severity of airflow limitation,^†,^^‡^ n (%)							
Moderate	111 (60.0)	118 (62.1)	178 (65.9)	184 (68.7)	58 (68.2)	108 (63.2)	104 (66.2)
Severe	72 (38.9)	68 (35.8)	92 (34.1)	84 (31.3)	27 (31.8)	63 (36.8)	53 (33.8)
≥1 COPD exacerbation in previous year,^‡^ n (%)	52 (28.1)	43 (22.6)	88 (32.6)	97 (36.2)	19 (22.4)	61 (35.7)	47 (29.7)
Concomitant use of ICS, n (%)	70 (37.6)	81 (42.6)	145 (53.1)	128 (47.6)	36 (42.4)	82 (48.0)	67 (42.4)

*At screening visit.

^†^Moderate COPD: 50% ≤postbronchodilator FEV_1_ <80% predicted and FEV_1_/FVC <0.70; severe COPD: 30% ≤postbronchodilator FEV_1_ <50% predicted and FEV_1_/FVC <0.70.

^‡^Patients with available data.

COPD, chronic obstructive pulmonary disease; FEV_1_, forced expiratory volume in 1 s; FVC, forced vital capacity; ICS, inhaled corticosteroid; ITT, intent-to-treat.

### Safety and tolerability

Safety and tolerability outcomes have previously been reported for each study.^17–19^ In summary, aclidinium is well tolerated with the most common adverse events being nasopharyngitis, headache, COPD exacerbation and cough. No clinically significant differences in other safety assessments were observed. No new safety and tolerability findings were anticipated based on these additional analyses.

### Daily COPD symptoms

**Figure 1:**
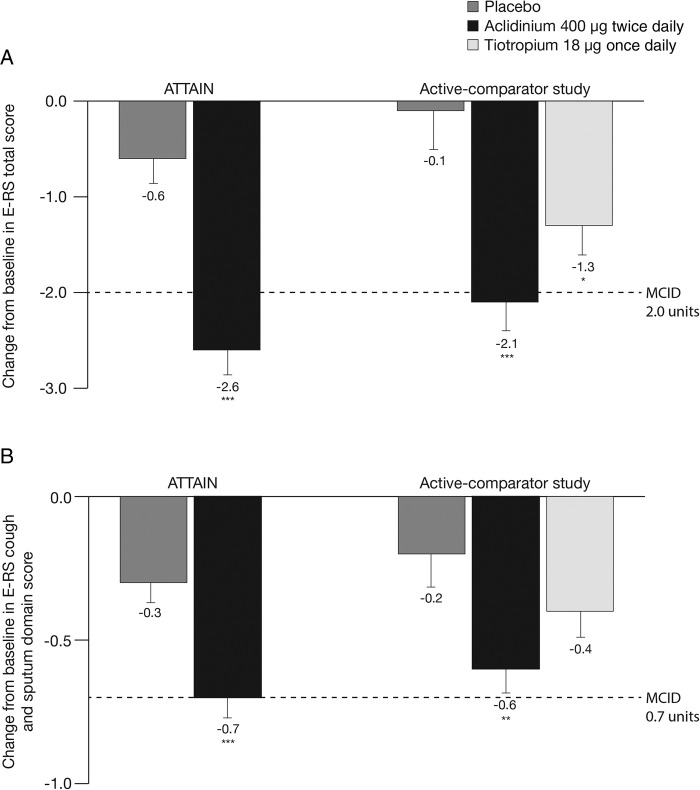
Change from baseline in (A) E-RS total score and (B) E-RS cough and sputum domain score over the study period in ATTAIN and the active-comparator study. Data are reported as LS mean+SE. E-RS total score ranged from 0 to 40; E-RS cough and sputum domain score ranged from 0 to 11. Higher scores indicate more severe symptoms. *p<0.05, **p<0.01, ***p<0.001 vs placebo. E-RS, Evaluating Respiratory Symptoms, formerly known as EXAcerbations of Chronic pulmonary disease Tool; LS, least squares; MCID, minimum clinically important difference.

Treatment with aclidinium 400 µg significantly reduced total daily COPD symptoms compared with placebo, as assessed by E-RS total score over 24 weeks in ATTAIN (p<0.001; figure 1A) and 6 weeks in the active-comparator study (p<0.001; figure 1A).^19^ In the active-comparator study, E-RS total score was also significantly reduced with tiotropium compared with placebo (p<0.05; figure 1A).

Daily cough and sputum symptoms, assessed by E-RS cough and sputum domain score, were also significantly reduced with aclidinium 400 µg versus placebo in ATTAIN and the active-comparator study (both p<0.01; figure 1B). There was no significant difference between tiotropium and placebo treatments on cough and sputum symptoms in the active-comparator study (p=0.109; figure 1B).

Post hoc analysis of a patient subpopulation with ≥1 exacerbation event identified by the EXACT (n=178) indicated that E-RS total score and E-RS cough and sputum scores were similar at baseline between aclidinium 400 μg and placebo in ATTAIN. After 24 weeks, treatment differences (95% CI) between aclidinium 400 μg and placebo in this group were significant for E-RS total score (−1.9 [−3.1 to −0.6]; p<0.01) and E-RS cough and sputum scores (−0.5 [−0.9 to −0.2]; p<0.01).

### Morning and night-time cough and sputum symptoms

**Figure 2:**
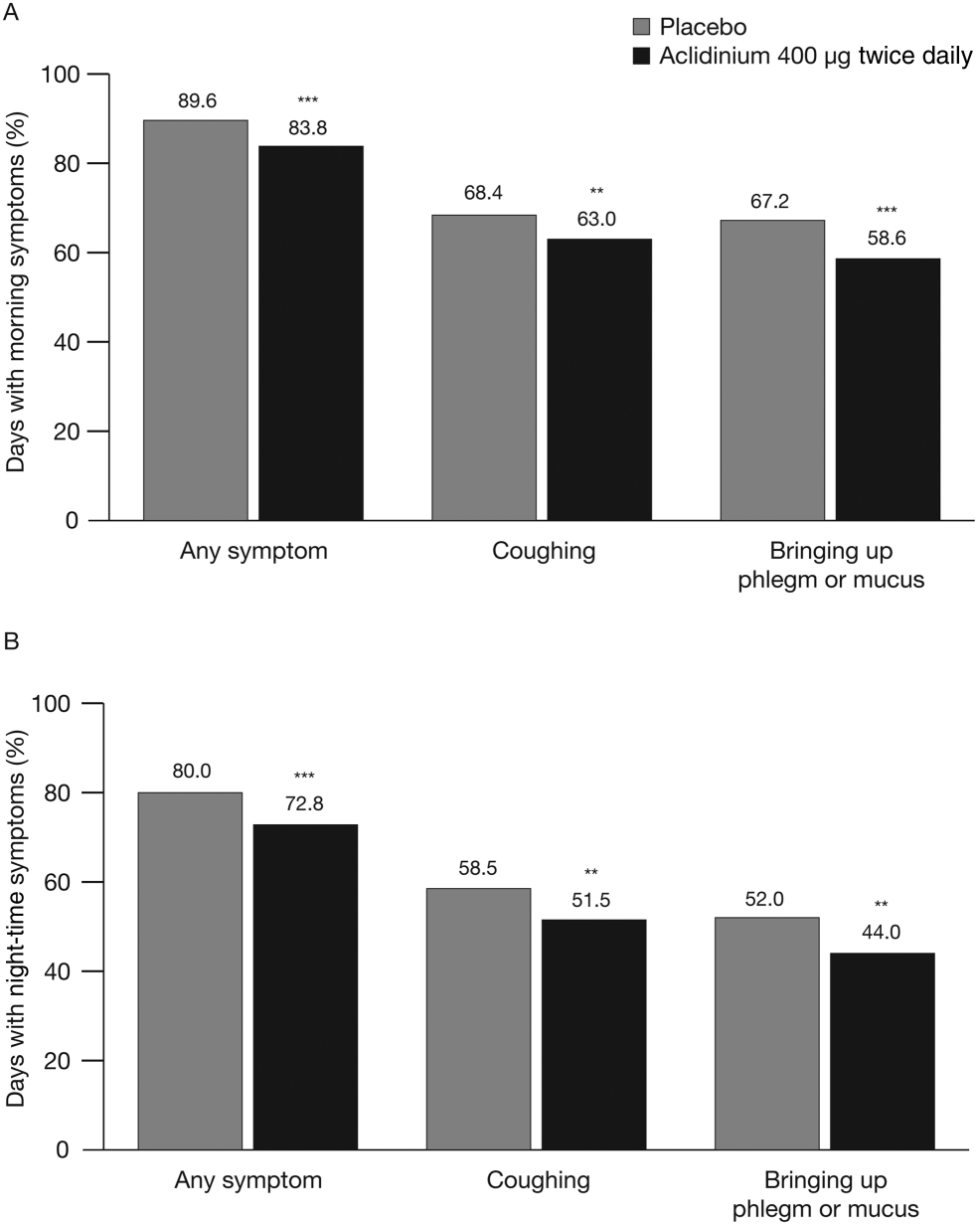
Percentage of days with (A) morning symptoms and (B) night-time symptoms over the study period in ATTAIN. Data are reported as least squares mean. **p<0.01, ***p<0.001 vs placebo.

In ATTAIN, the percentage of days with any morning or night-time symptoms over the study period was significantly lower in patients treated with aclidinium 400 µg compared with placebo (both p<0.001; figure 2). Aclidinium treatment also significantly reduced the percentage of days with morning or night-time cough symptoms compared with placebo (both p<0.01; figure 2). Similarly, the percentage of days with morning or night-time bringing up phlegm or mucus was also significantly lower over the study period in patients treated with aclidinium 400 µg compared with placebo (p<0.01; figure 2).

In the active-comparator study, both aclidinium and tiotropium significantly increased the change from baseline in the percentage of days without any morning symptoms over the study period versus placebo (treatment differences [95% CI] 8.9% [4.1% to 13.8%] with aclidinium and 5.6% [0.6% to 10.6%] with tiotropium; p<0.001 and p<0.05 vs placebo, respectively). Similarly, there was a significant increase in the percentage of days without morning cough symptoms in the aclidinium group compared with placebo (treatment difference [95% CI] 7.2% [1.1% to 13.4%]; p<0.05); there was no significant difference with tiotropium versus placebo (treatment difference [95% CI] 5.5% [−0.8% to 11.8%]; p=0.084). While the change from baseline in the percentage of days without difficulty bringing up phlegm was numerically higher with aclidinium (7.7%) and tiotropium (4.8%) compared with placebo (2.0%), the differences between the active treatments and placebo did not reach statistical significance (p=0.100 for aclidinium and p=0.425 for tiotropium).

**Figure 3:**
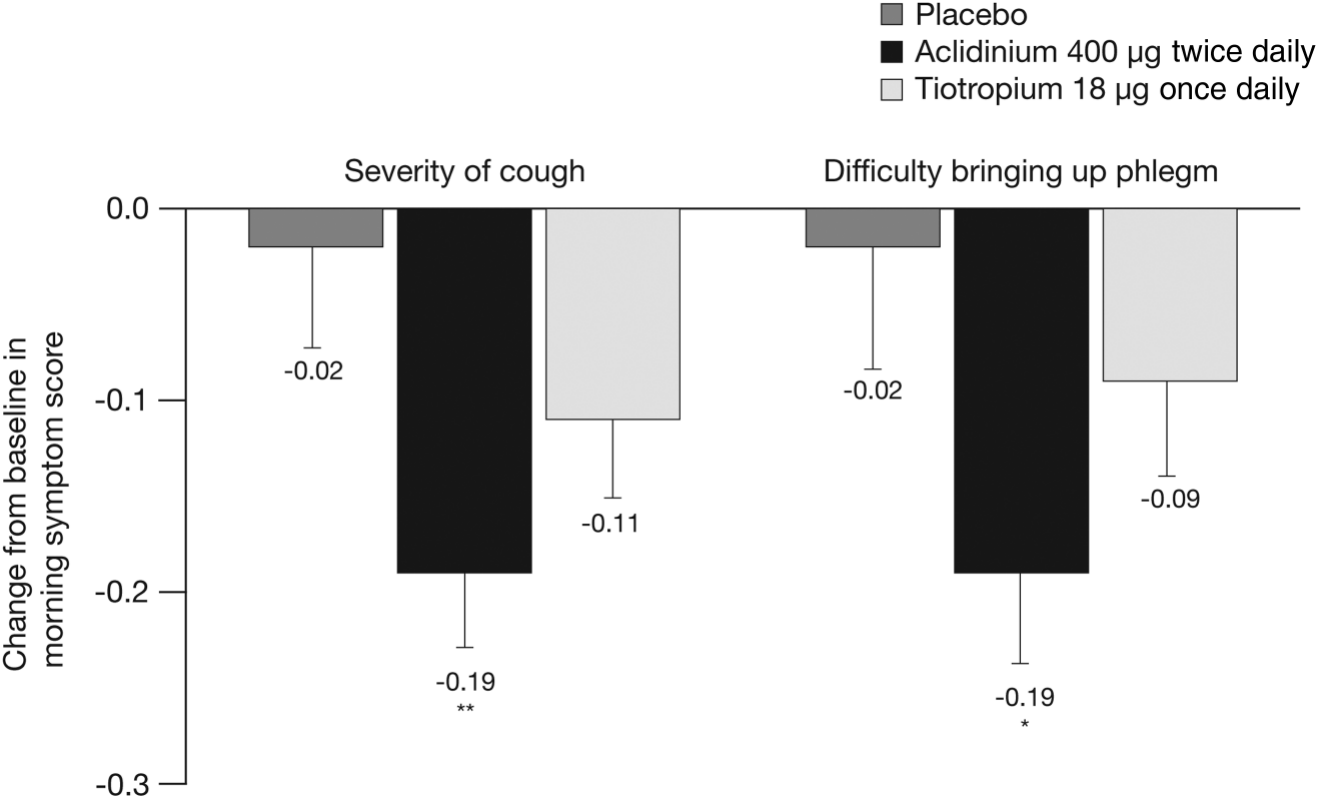
Change from baseline in the severity of morning cough and severity of difficulty bringing up phlegm in the morning over the study period in the active-comparator study. Data are reported as LS mean+SE. Assessed on a 5-point scale: 0=‘no symptoms’ to 4=‘very severe symptoms’. *p<0.05, **p<0.01 vs placebo. LS, least squares.

Patients' assessment of the overall severity of their morning symptoms over the study duration was significantly reduced with aclidinium (−0.22; p<0.001) and tiotropium (−0.12; p<0.05) compared with placebo in the active-comparator study.^19^ When the severity of morning cough and difficulty bringing up phlegm was assessed, there was a significant reduction in the severity of both symptoms with aclidinium versus placebo over 6 weeks (p<0.05; figure 3). There was no significant change from baseline in the severity of either cough or difficulty bringing up phlegm in patients treated with tiotropium compared with placebo.

**Figure 4:**
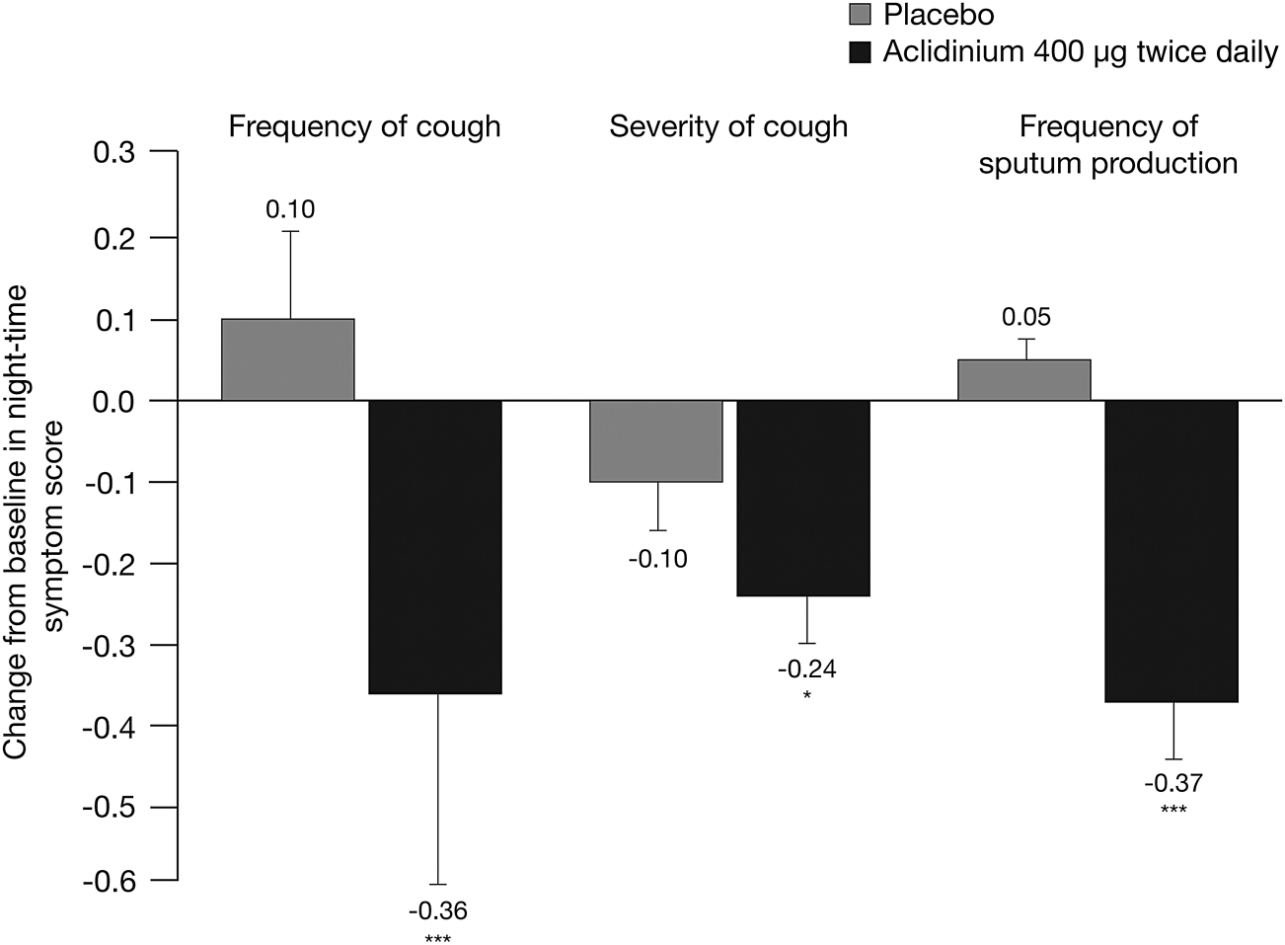
Change from baseline in the severity of night-time cough and the frequency of night-time sputum production at week 12 in ACCORD COPD I.^17^ Data are reported as mean+SE. Symptom frequency assessed on a 5-point scale: 0=‘never’ to 4=‘7 or more times’. Symptom severity assessed on a 5-point scale: 0=‘no symptoms’ to 4=‘severe symptoms that interfered with normal daily activities’. *p<0.05, ***p<0.001 vs placebo.

ACCORD COPD I investigated the prevalence and severity of night-time cough and sputum symptoms.^17^ After 12 weeks of treatment, aclidinium 400 µg significantly reduced the frequency of night-time cough compared with placebo (p<0.001; figure 4). The severity and impact of night-time cough symptoms was also significantly reduced at week 12 with aclidinium 400 µg compared with placebo (p<0.05; figure 4). In addition, the frequency of night-time sputum production was significantly lower in patients treated with aclidinium 400 µg compared with placebo (p<0.001; figure 4).

### Correlation between E-RS and symptom questionnaires

When all treatment groups were combined, there was significant correlation between the improvement in E-RS cough and sputum domain score and the improvement in the severity of morning cough symptoms assessed using the symptom questionnaire (r=0.684; p<0.001). Similar results were observed when the correlation between scores was assessed in each active treatment group (data not shown).

## Discussion

This analysis is the first to specifically investigate the impact of a LAMA, or indeed any bronchodilator, on cough and sputum symptoms in patients with stable moderate-to-severe COPD. The results of the ATTAIN, ACCORD COPD I and active-comparator studies provide evidence that aclidinium is effective at reducing the severity and frequency of cough and sputum symptoms in patients with COPD, with improvements in E-RS total and E-RS cough and sputum scores as well as in evaluations of morning and night-time symptoms. These improvements were seen throughout the day and irrespective of the assessment tools used. Furthermore, aclidinium has previously been shown to be well tolerated in patients with COPD.^17–19^ ^23^

Chronic cough and mucus accumulation in the airways are strongly associated with disease progression, lung-function decline and risk of adverse outcomes in patients with COPD.^2–4^ However, most clinical trials designed to evaluate the efficacy of COPD treatments have focused on improvements in lung function and breathlessness and reductions in exacerbation risk as clinical outcomes. The few trials that have assessed the efficacy of a LAMA on cough and sputum symptoms to date have reported negative results. In phase III studies, there was no significant difference in physicians' assessment of cough symptoms between patients receiving tiotropium and those receiving placebo.^32^ ^33^ Furthermore, no effect on mucociliary clearance was observed with ipratropium or tiotropium in patients with COPD.^34^ ^35^ In contrast, patients with severe COPD treated with an inhaled corticosteroid (fluticasone) and a long-acting β_2_-agonist (salmeterol) have been shown to report significantly reduced cough symptoms versus placebo.^36^ Before the current analysis, only smoking cessation has consistently been shown to reduce cough and phlegm symptoms in patients with COPD.^37^ ^38^

Patients with COPD report variability in the frequency and severity of cough and sputum symptoms throughout the day, with greatest impact first thing in the morning and at night-time.^10^ ^11^ ^39^ The prevalence and severity of cough symptoms at the start of the day may relate to periods of increased activity associated with getting washed and dressed, whereas the night-time cough and sputum symptoms may be a consequence of mucus hypersecretion or reduced ciliary activity. In the analyses reported here, treatment with aclidinium significantly reduced the percentage of days and nights with symptoms of coughing and bringing up phlegm or mucus. The severity of morning and night-time cough and sputum symptoms was also found to be reduced. Treatment approaches that impact on cough and sputum symptoms throughout the whole 24-hour day may provide clinical benefits to some patients in terms of their overall well-being, particularly in the morning and night-time when patients report that these symptoms are most troublesome.

In the active-comparator study, while both LAMAs had an effect, the magnitude of improvement in overall symptoms and cough and sputum symptoms was greater with aclidinium compared with tiotropium. The reasons for this are unclear; however, the fact that both LAMAs improved symptoms of cough and sputum suggests that these may be class effects. Precisely how these compounds might exert an effect on cough and sputum is unclear, but there is an emerging body of preclinical evidence suggesting that multiple pathways may be involved. For example, there is evidence that muscarinic antagonists reduce experimental cough,^40^ and that tiotropium and ipratropium act on TRPV1 to reduce the cough response in preclinical models.^41^ In addition, a 2016 preclinical study in rabbits showed that, further to their anticholinergic activity and any action on TRPV1 receptors, aclidinium and tiotropium may also have antitussive actions involving mechanoreceptors and acid-sensing ion channels.^42^ These studies provide preclinical evidence of LAMA antitussive activity; however, it is not yet clear how this may translate into clinical practice.

Studies of capsaicin responsiveness suggested an increased cough reflex in patients with COPD;^43^ however, a recent study which evaluated predictors of cough frequency found no significant relationship between cough frequency and capsaicin cough reflex sensitivity.^39^ In contrast, cough frequency was independently associated with being a current smoker, smoking history, sputum production and neutrophilic inflammation.^39^ A recent study has demonstrated that M3 receptors may play a proinflammatory role in cigarette smoke-induced inflammation in animal models of COPD, suggesting another potential mechanism by which LAMAs may improve cough in patients with COPD.^44^ This is further supported by preclinical studies that have shown LAMAs can reduce neutrophils and inflammatory mediators, such as interleukin-6, tumour necrosis factor-α and interferon-γ, in cigarette smoke-exposed animal models.^45^ ^46^ The efficacy of LAMAs to improve cough and sputum symptoms requires further investigation to determine if the effects observed with aclidinium are also seen with other drugs in this class.

The E-RS and night-time symptoms of COPD questionnaires are validated tools for assessing cough and sputum symptoms in patients with COPD.^24^ ^28^ The observed improvements in E-RS cough and sputum symptoms with aclidinium 400 µg in ATTAIN (0.7 decrease from baseline in 24 weeks) and the active-comparator study (0.6 decrease from baseline in 6 weeks) compare well with the recently proposed minimum clinically important difference (MCID) of ≥0.7 decrease from baseline.^47^ The lack of a validated MCID in the other tools used in these studies may be considered to be a potential limitation of this analysis. However, the significant correlation between improvements from baseline in E-RS cough scores and the severity of morning cough symptoms assessed using symptom questionnaires in the active-comparator study supports the clinical utility of these tools to assess cough symptoms.

This study has other potential limitations. It should be stated that none of the three phase III studies reported here was powered to detect differences in cough and sputum symptoms, and the studies were not specifically designed to assess these symptoms. Furthermore, there was no prespecified minimum level of symptoms in any of the studies, meaning the population was relatively heterogeneous in terms of symptoms. Clinical trials designed specifically to assess the effects of treatments on cough and sputum symptoms in patients with COPD, using a combination of patient-reported outcomes, cough-specific quality-of-life measures and objective measures of cough and sputum symptoms, are needed to fully understand the efficacy of novel treatments on these symptoms.

## Conclusions

While few studies have investigated the effect of bronchodilators on cough and sputum symptoms, the results reported here suggest that in addition to improving lung function, LAMAs, such as aclidinium, can improve cough and sputum expectoration compared with placebo in patients with COPD. As cough and sputum symptoms impact negatively on overall patient well-being, controlling these symptoms may represent an important additional therapeutic benefit of this class of drugs.

## Footnotes

i Registered trademarks of AstraZeneca group of companies; for use within the USA as Pressair^®^ and Genuair^TM^ within all other licensed territories.
