# Supplementary material for: Effect of aclidinium bromide on cough and sputum symptoms in moderate-to-severe COPD in three phase III trials
Source: BMJ Open Respir Res. 2016 Dec 8;3(1):e000148. doi: 10.1136/bmjresp-2016-000148 (PMC5174811; doi:10.1136/bmjresp-2016-000148)
Supplement: supplementary tables [file bmjresp-2016-000148supp_tables.pdf]

Supplementary Table S1. Summary of patient-reported outcome measures used to capture symptoms in each clinical trial and corresponding endpoints

	ATTAIN	ACCORD COPD I	Active-comparator study	Endpoints
**EXACT E-RS**				
Total score	X		X	Change from baseline in E-RS total score
Cough and sputum domain	X			Change from baseline in E-RS cough and sputum domain
**SYMPTOM QUESTIONNAIRES**				
6-item nighttime and morning symptoms of COPD questionnaire	X			Percentage of days with morning or nighttime symptoms
9-item COPD morning symptoms questionnaire			X	Change from baseline in: % days without morning symptoms; severity of morning cough; difficulty bringing up phlegm
11-item COPD nighttime symptoms questionnaire		X		Change from baseline in COPD nighttime symptoms

Supplementary Table S2. Baseline E-RS scores, E-RS cough and sputum domain score, nighttime symptoms questionnaire, nighttime and morning symptoms of COPD questionnaire and COPD questionnaire results in ACCORD COPD 1, ATTAIN and active-comparator studies

	**ACCORD COPD I**		**ATTAIN**		**Active-comparator study**		
	**Placebo (N=185)**	**Aclidinium 400 µg BID (N=190)**	**Placebo (N=273)**	**Aclidinium 400 µg BID (N=269)**	**Placebo (N=85)**	**Aclidinium 400 µg BID (N=171)**	**Tiotropium 18 µg QD (N=158)**
E-RS scores[a]							
Total, mean (SD)			13.6 (6.6)	14.1 (6.4)	13.4 (6.0)	13.6 (5.8)	12.5 (5.8)
Cough and sputum domain, mean (SD)			3.8 (1.9)	3.9 (1.8)	3.7 (1.8)	3.9 (1.6)	3.6 (1.5)
COPD nighttime symptoms questionnaire							
Frequency of nighttime cough[b] mean (SD)	2.06 (1.50)	1.92 (1.61)					
Frequency of nighttime sputum production[b] mean (SD)	1.33 (1.43)	1.35 (1.55)					
Severity and impact of nighttime cough[c] mean (SD)	1.49 (0.88)	1.39 (0.97)					
Nighttime and morning symptoms of COPD questionnaire							
Mornings with any COPD symptoms,[d] %, mean (SD)			89.3 (22.6)	89.5 (23.2)			
Mornings with any cough symptoms,[d] %, mean (SD)			70.8 (34.2)	68.9 (37.0)			
Mornings with any bringing up phlegm or mucus symptoms,[d] %, mean (SD)			63.9 (36.6)	64.9 (37.7)			
Nights with any COPD symptoms,[d] %, mean (SD)			84.2 (26.4)	84.0 (27.3)			
Nights with any cough symptoms,[d] %, mean (SD)			65.3 (35.3)	63.2 (37.5)			
Nights with any bringing up phlegm or mucus symptoms,[d] %, mean (SD)			56.0 (37.1)	57.7 (37.1)			
COPD symptoms questionnaire							
Days without morning COPD symptoms,[e] %, mean (SD)					10.2 (22.3)	11.1 (23.1)	14.0 (24.9)
Days without morning cough symptoms,[e] %, mean (SD)					27.2 (34.3)	27.1 (33.7)	30.5 (35.9)
Days without morning difficulty bringing up phlegm symptoms,[e] %, mean (SD)					68.9 (38.5)	59.9 (37.3)	69.7 (35.7)
Overall severity of morning symptoms,[f] mean (SD)					2.30 (0.61)	2.36 (0.61)	2.25 (0.62)
Severity of morning cough symptoms,[f] mean (SD)					1.20 (0.76)	1.29 (0.74)	1.15 (0.73)
Severity of morning difficulty bringing up phlegm symptoms,[c] mean (SD)					0.62 (0.82)	0.86 (0.88)	0.60 (0.78)

[a]E-RS total score ranged from 0 to 40; E-RS cough and sputum domain score ranged from 0 to 11. Higher scores indicate more severe symptoms

[b]Assessed on a 5-point scale: 0 = ‘never’ to 4 = ‘7 or more times’
[c]Assessed on a 5-point scale: 0 = ‘no symptoms’ to 4 = ‘severe symptoms that interfered with normal daily activities’
[d]Assessed as the average proportion of days during the run-in period
[e]Assessed as the average proportion of days during the run-in period
[f]Assessed on a 5-point scale: 0 = ‘no symptoms’ to 4 = ‘very severe symptoms’
BID, twice daily; COPD, chronic obstructive pulmonary disease; E-RS, Evaluating Respiratory Symptoms, formerly known as EXAcerbations of Chronic pulmonary disease Tool; ITT, intent-to-treat; QD, once daily; SD, standard deviation
